# Parents' experiences of an abnormal ultrasound examination - vacillating between emotional confusion and sense of reality

**DOI:** 10.1186/1742-4755-7-10

**Published:** 2010-06-14

**Authors:** Anna-Karin Larsson, Elizabeth Crang Svalenius, Anita Lundqvist, Anna-Karin Dykes

**Affiliations:** 1 Faculty of Medicine, Department of Health Sciences, Division of Nursing, Lund University, PO Box 157, SE-221 00 Lund, Sweden; 2 Department of Obstetrics and Gynecology, Lund University Hospital, SE- 221 85 Lund, Sweden

## Abstract

**Background:**

An ultrasound examination is an important confirmation of the pregnancy and is accepted without reflection to any prenatal diagnostic aspects. An abnormal finding often comes unexpectedly and is a shock for the parents. The aim was to generate a theoretical understanding of parents' experiences of the situation when their fetus is found to have an abnormality at a routine ultrasound examination.

**Methods:**

Sixteen parents, mothers and fathers, whose fetus had been diagnosed with an abnormality during an ultrasound scan in the second or third trimester, were interviewed. The study employed a grounded theory approach.

**Results:**

The core category *vacillating between the emotional confusion and sense of reality *is related to the main concern *assessment of the diagnosis impact on the well-being of the fetus*. Two other categories *Entering uncertainty *and *Involved in an ongoing change and adaptation *have each five sub-categories.

**Conclusions:**

Parents are aware of that ultrasound examination is a tool for identifying abnormalities prenatally. The information about the abnormality initially results in broken expectations and anxiety. Parents become involved in ongoing change and adaptation. They need information about the ultrasound findings and the treatment without prolonged delay and in a suitable environment. The examiner who performs the ultrasound examination must be aware of how anxiety can be intensified by environmental factors. All parents should to be offered a professional person to give them *s*upport as a part of the routine management of this situation.

## Background

Major fetal abnormalities are often detected by a prenatal ultrasound examination [1]. According to a Swedish population-based study from 2006, the prevalence of diagnosed malformations was 2.6% with one routine ultrasound screening [2]. Levi [3] showed in a review of available studies that in specialist centres the detection rate of fetal abnormalities ranges from 80% to 95%. When the ultrasound examination reveals abnormal findings, it is a shock for the parents-to-be as found in a systematic review from 2002 [4]. Women often experience emotional reactions such as anxiety, prostration, depression, and loneliness [5], as well as guilt, fear, sadness or hopelessness. Women also begin grieving at the time of the fetal ultrasound examination while men experience anger and anxiety [6] and both of the parents experience the emotions of anxiety, isolation and insecurity [7]. This also raises thoughts about the loss of their imagined future, through having no child or a handicapped child which would require readjustment by the entire family [8]. It seems to be common to feel increased anxiety while waiting for the ultrasound test result or after receiving information revealing an abnormal finding [7,9].

In general, parents have a very positive attitude towards an ultrasound examination and consider it as a natural and important part of their maternity care and pregnancy and very few decline, the offer being a 'channel of desire' [10,11]. The examination has a social meaning for the parent's, they do not reflect upon the examination as prenatal diagnosis and are therefore not prepared for any negative results the examination can give [12]. Ultrasound examinations have a strong beneficial psychological effect on the pregnant woman and her partner in reassuring them about their pregnancy [13,14]. It is a way of meeting the unborn child [15-18] and a confirmation that the expected child is healthy [15]. Mitchell [18] also found that getting information and an image of the fetus appearance sometimes gave the women a feeling of being a mother. Generally this becomes the focus of the parents, not the medical purpose i.e. screening for structural anomalies or to exclude malformations [2]. Anxiety during pregnancy can never be totally prevented because it is a natural feeling to experience during this special period in life [7]. If the fetus is diagnosed with an abnormality, and if the parents decide to continue the pregnancy, this knowledge of course influences their experience of the pregnancy and daily life. In such situations it is important to prepare the parents for the birth and management of the care that the child may need [19-21]. Swedish women have a right to decide about termination of pregnancy before completing the 18^th ^week of gestation. After the 18^th ^week of pregnancy termination is only permitted when special reasons exist and must be approved by the Swedish Health Authorities [22]. However, it cannot be assumed that accepting an offer of screening implies that parents are willing to terminate the pregnancy if a fetal defect is detected [20].

Swedish recommendations are to offer all pregnant women an ultrasound examination at 16 to 20 weeks' gestation and possibly a second examination at 32 weeks' gestation [23]. A normal pregnancy and delivery is the midwife's prerogative and most routine ultrasound examinations are preformed by midwives. When detecting any abnormality during the examination, it ceases to be normal and the routine is that a physician has to be contacted as a midwife is not permitted to inform about a pathological diagnosis (ibid). Similarly routines are practiced in other countries [24,25]. A previous quantitative study involved a part about parents' experience of the ultrasound examination situation. The result according to PEER-U, State of Mind Index (an ultrasound-specific instrument) showed that if the ultrasound finding is abnormal, the parents experienced the examiner as less personal and they also experienced that they were given less information about what could be seen during the examination. Furthermore, they felt that they, to a lesser degree, could ask questions during the examination. Probably the parents had these experiences because the midwife is responsible for the normal ultrasound and should not discuss adverse findings and diagnosis. The gender analysis showed that both men and women thought; they were given less information during the scan about what could be seen; they felt to a lesser extent a sense of security during the scan; they felt to a lesser extent well informed during the scan and finally they felt to a greater extent worried despite the fact that they could understand the ultrasound image [26]. Mitchell [18] examined how 42 women perceived ultrasound when they received unexpected abnormal ultrasound findings. She found that it was not always that the sonographer told about an unexpected finding but in spite of that the women became suspicious because they found that the scan was taking too long, looking at one part of the fetus or that the sonographer's behaviour changed from carrying on a conversation to being quiet or frustrated ([18] p 230, [7]). When the parents accidentally found out by themselves that something was wrong with the fetus the women was dismayed and confused ([18] p 231). Lalor et al [24] published an article relating the experiences of women in Eire from their encounters with caregivers after diagnosis of fetal abnormality following the second ultrasound scan. The women involved wanted information from the fetal medicine specialist as soon as possible and written information was seen as essential to ensure understanding. Continuity of caregiver as well as empathy from staff was strongly valued [19]. To sum up, the reviewed literature shows that parents, in connection with an ultrasound examination, are not prepared for the findings to be abnormal. There is only sparse literature describing both parents' experiences when dealing with an unexpected ultrasound finding. The aim of this study was to generate a theoretical understanding on how both parents' can experience the situation when their fetus is found to have a fetal abnormality at a routine second or third trimester ultrasound scan.

## Methods

The participants were derived from a one year cohort study which included all parents who had their second trimester ultrasound examination at a Swedish University hospital during the period February 2005 to March 2006. All staff was aware of the cohort study and the interview studies it could generate. However, they did not know which parents would be interviewed. At this department a third trimester ultrasound was also offered as a routine. The scans were performed by specially trained midwife sonographers, with a 'back-up' physician for consultation when findings were doubtful or abnormal. For this study a theoretical sampling was chosen in accordance with grounded theory, which means that the data collection is based on the concepts developed in the memos. Inclusion criteria were Swedish speaking pregnant women and their partners whose fetus had a deviation from normal at the ultrasound scan in the second or third trimester. In the current study all partners were fathers to the expected child. Abnormal ultrasound findings of isolated soft markers were excluded.

Totally nine couples (mothers and fathers) were invited to participate in the study and all but two fathers participated. One of these fathers had not been present at the ultrasound examination and was not living with the mother any longer. The other father was unable to attend the interview. The parents had already given their written consent by answering a questionnaire about their experience of the second trimester routine ultrasound concerning normal and abnormal findings, reactions and needs, which also included the collecting of demographics [26-28]. The parents were contacted by phone and asked to participate in this study. A time for the interview was arranged after receiving the parents' verbal consent.

The interviews took place from 2005 to 2008. The interviews began with the open question "Can you tell me about your experiences of the second or third trimester ultrasound examination, starting from your arrival at the ultrasound unit, where the abnormality was found". This question was followed up with further questions e.g. please clarify, what did you feel/think, in order to deepen the narrative and increase the understanding. The interviews took on average 60 minutes. A time was reserved for conversation before and after the interview. The first author conducted all but two of the interviews, one midwife and one paediatric nurse did the other two interviews. Ethical approval was obtained from the Research Ethics Committee of the Medical Faculty of the University of Lund, Sweden (LU-453-00).

The study employed a grounded theory approach according to Glaser [29]. Grounded theory [30] is a suitable method for behavioural research in a social context. The method is both inductive in the way of starting with an open question and open coding followed by developing hypothesis. It is deductive when creating specific questions from the developed hypothesis and performing the theoretical coding. Grounded theory is a qualitative design recommended to researchers who want to generate a theory from data that is systematically collected through in-depth interviews and analysed using constant comparative method (ibid).

In grounded theory collecting the data and analysing the material is a parallel process. The interviews were tape-recorded, de-identified and transcribed verbatim. The analysis of the data began directly after the first interview with the purpose of conceptualising the material. This interactive process continued after each interview as a constant comparative analysis in order to systematically approach the data [29]. Memos (thoughts and reflections arising when reading each interview leading to ideas) were made after each interview and used towards the end of the analysis. In the next stage, the open coding, line by line analysis was performed and each interview generated several substantive codes. These were compared, some of them were grouped together but also new ones were generated, categories explaining the content emerged. In the next stage the categories were collapsed to find the abstract level and to find the core category that was related to the other categories as well as to the main concern - the basic social psychological processes i.e. "the assessment of the impact of the diagnosis on the fetus well-being". In the next phase only codes relevant for the core category were analysed. The categories were then analysed in what way they could be interrelated as hypotheses in order to be integrated into the model/theory [29].

## Results

Parents (nine different pregnancies, nine mothers and seven fathers n = 16)) of fetuses with different abnormalities (Table 1), identified at an ultrasound examination in the second or third trimester, were interviewed. Six couples and one mother were interviewed approximately one year after giving birth and the other two interviews took place two months after the termination or IUFD. In these two interviews the partner also participated in one. When both parents participated they were interviewed together, at their own request. All parents lived in the south of Sweden, had been scanned in the same hospital department and all but one had Swedish background, one woman came from Asia. The women's ages, at the time for the child's birth or termination of pregnancy, was between 26 to 36 years (mean 31 years). Four of the women were nulliparous, four expected their second child and one her third child. One woman had had an abnormal ultrasound examination earlier in a prior pregnancy. Three of the fetuses had one or more additional abnormality that was discovered after termination or at birth.

**Table 1:** The abnormalities found at a second or third trimester ultrasound

**Interview No**.	Ultrasound finding
1.	Induced abortion due to kidney dysplasia (*lethal*) choroid plexus cysts and other small deviations

2.	IUFD - Trisomy 18 (*lethal*)

3.	Fetal hydronefrosis - Dilated renal pelvis(*treatable*)

4.	Multiple malformations, hypophysis insufficiency, cardiac anomaly, extra finger, choroid plexus cysts (*treatable with and without sequele*)

5.	Bilateral pes equinovarus (club foot)(*treatable*)

6.	Cardiac anomaly (*treatable, can leave sequele*)

7.	Duodenal atresia (double bubble)(*treatable*)

8.	Cleft lip (*treatable with good results*)

9.	Omphalocele, heart malplacement, echogenic focus in the right ventricle (*treatable can leave sequele*)

**Figure 1:**
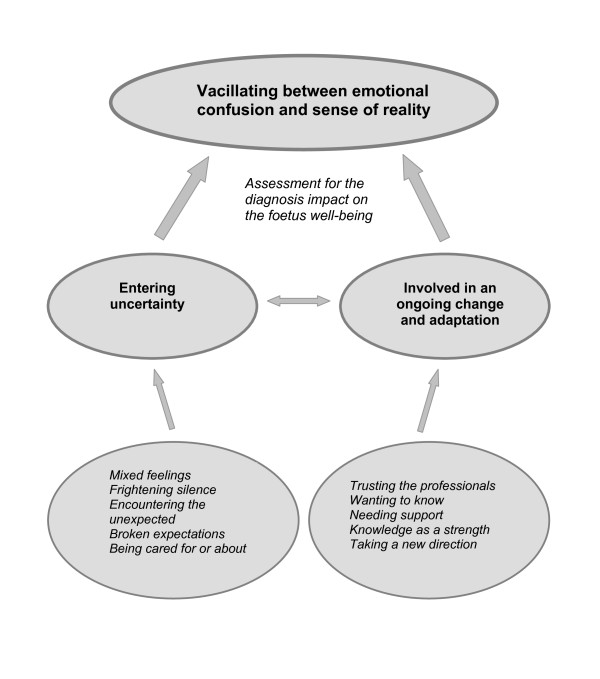
Generated theoretical model of parents experiences of the ultrasound situation when their fetus was found to have an abnormality.

In total, thirteen concepts in relation to the parent's experiences of an ultrasound examination emerged from the data and one category dominated. This category was named "*vacillating between emotional confusion and sense of reality" *and became the core category. It is related to the main concern, "the assessment of the impact of the diagnosis on the fetus well-being", and gives an understanding as to how the parents struggled with their feelings when they were informed about the fetal abnormality. It is also related to the other two categories with subcategories in being the overall category which has more comprehensive meaning than the others. Two categories emerged: "Entering uncertainty" with five sub-categories (Mixed feelings; Frightening silence; Encountering the unexpected; Broken expectations; Being cared for or about) and "Involved in an ongoing change and adaptation" with the following five sub-categories (Trusting the professionals; Wanting to know; Needing support; Knowledge as a strength; Taking a new direction) (Figure 1).

### Vacillating between emotional confusion and sense of reality

When the parents were informed of the abnormalities found with ultrasound they were often very distressed. Some parents were informed of the abnormality at once and some were only simply informed that something was wrong with the fetus. *"When coming out [from the ultrasound examination] one was quite confused. The coming days I was in low spirits, it [the abnormality] circulated in my head the whole time and I could not do anything" (mother 9)*. Irrespective of this, the parents hovered between the knowledge that the fetus had some abnormalities and the hope that the fetus was normal i.e. healthy. *I became very distressed when they told me about the malformation, but they said he would be alright after the operation (mother 6)*. In this way the parents retained their common sense and felt a sense of security in that their fetus was a "little bit abnormal", or that it was possible to adjust or treat after birth. Many parents noted that the staff had left them with the impression that they felt that the parent's reactions to the information and their level of distress was often out of proportion to the seriousness of the abnormality. "*I think the toughest thing for us was that they compared us with parents who had it worse; our thing was a trifling find. That was hard because if you find something that is wrong, it is supposed to be corrected, then it is for real and I don't care about the others" (father 9)*. The parents adjusted to the circumstances during the ultrasound examination, trusting the professionals and accepted the treatment though felt ignored concerning the information and the fact that their anxiety was not taken seriously. They lived with a fear but tried to take control of their senses. The parents vacillated between the emotional confusion with the desperate thought of a sick child and the sense of reality of getting a child to take care of. *"Well I think it [ultrasound examination] is a torment, from beginning to end" *(mother 1).

## Entering uncertainty

This category concerns one of the strong feelings among the parents that influenced their experience of receiving bad news during the ultrasound examination and has five sub-categories.

### Mixed feelings

None of the parents had thought of not accepting the offer of the ultrasound examination. All had feelings of anxiety already when entering the ultrasound department. Some of them had earlier experiences of ultrasound examinations, and therefore knew what to expect, or they thought they knew. The expectation that the ultrasound examination created was mostly explained as a positive feeling but for some as a negative feeling. All parents knew that ultrasound was a tool for identifying abnormalities, although for most of them, this was not the main purpose of the examination. *We had thought about that something could be wrong, but still you don't expect it (mother 2)*. This was a time for confirmation of the pregnancy and a first opportunity to meet the expected child. *We were full of expectation and thought it would be very exciting to see our baby (Father 4)*. One couple did not think of an ultrasound examination as a chance to see their child, but rather simply as fetal diagnosis and nothing else.

### Frightening silence

The ultrasound examination was performed in silence in a half-dark room and only the droning sound from the ultrasound machine was heard. This was to some extent frightening for the parents. In this silence they noted a sense of seriousness. The quiet environment intensified the anxiety they felt when they first entered the room even if they could understand why the silence was important for the examination. Also their time perception changed, a couple of minutes now felt like hours. Another experience commented on was the body language of the person performing the scan and how the parents interpreted this. *He [the physician] was concentrating on what he was supposed to do and I was laying there and becoming more anxious about everything and for every humming or so the anxiety increased and I thought, oh, what is it now, hasn't she got arms and legs either? (mother 9)*. They had an intuition that bad news was coming or at least that something was different from normal. *She [the midwife] was very nice, it wasn't her fault but very soon you could understand that something was wrong (mother 4)*. The parents felt uncomfortable with the situation but understood that concentration was needed during the examination, especially if something unexpected was found.

### Encountering the unexpected

As mentioned earlier, the parents felt that bad news was coming on the basis of how they interpreted the body language of the sonographer. However, even if both staff and parents were aware of that the examination could show an abnormality, the examination result was expected to be normal, as it generally is. Eventual bad news was delivered very differently from case to case. The parents appreciated honest information even if it caused them anxiety but, as they expressed, not knowing or not understanding causes even more anxiety. *She had written down clubfoot and we said ok, and then she [the midwife] said "but that is not such a severe illness" but we didn't know anything (mother 5)*. Sometimes parents thought that the sonographer felt uncomfortable when making an unexpected finding and that it affected how the news was given. *She [the midwife] said it as if it [the finding] was as clear as daylight but I think she thought the situation was horrible (mother 8)*. The parents also had the feeling that the midwife was not permitted to inform them about a finding and what to expect. *She said something that she was not allowed to tell, I think (mother 9).*

### Broken expectations

When it was a fact that the fetus had an abnormality the parents felt their expectations dashed. *You are very sad and shocked in the beginning, actually totally devastated because the dream of the perfect child is broken (mother 8)*. They had heard that something was wrong but were not able to understand the information given them because their feelings of anxiety overshadowed it. Their emotional state could cause them to be confused in their effort to understand the information because the parents wanted the examination to be normal. *Well, you so very much want everything to be alright that you fool yourself, because you want it so much (father 9)*. After receiving the bad news, it was difficult for them to tell relatives about what had happened. How the message was perceived was dependent on if the finding was operable and the predicted outcome. *I don't know but I think if you just hear that there is a possible treatment, you always feel very secure (father 3)*. In some cases the information from the sonographer caused the parents unnecessary anxiety because it was too brief and gave free scope to their imagination. *We were just about to buy a house with stairs and thought; is it better for us to break off the deal because it would never work if he [the child] is not able to walk (father 5).*

### Being cared for or about

A big part of the category "entering uncertainty" concerns how the parents experienced being in the situation. Feelings like being left out, ignored, feeling vulnerable, were feelings that did not give the parents any stability to manage it. However, the uncertainty from being in the situation also involved feelings like being part of, getting confirmation of and being listened to, making it possible for the parents to find some stability to manage their trying predicament. Different things contributed to the parents' feelings of uncertainty and the most common were environmental. The women and their partners told about how the women were laying down half-naked when the information, related to the findings, was presented. This position was acceptable when giving the preliminary information, but staying in this position during the whole time felt insulting and they felt at a disadvantage. The parents experienced a feeling of not being involved when the staff talked about "the baby" in an impersonal way apparently not recognizing them as the parents. In one case this feeling did not change until the parents came home after the baby's operation and hospital stay. *Now she was our daughter, in some way she hadn't been that before. We weren't important; she *[the child] *was (mother 9)*. When being involved in any discussion they had a positive attitude towards their experiences even if they were anxious. Feelings of empathy, being in good hands and being taken seriously increased their confidence. *When the care planning was done, we were sitting in an upright position, in daylight, so that we could see each other when talking (mother 1).*

## Involved in an ongoing change and adaptation

This second category with five sub-categories elucidates the parents' process of changing and adjusting to their experience and new life situation.

### Trusting the professionals

The parents' experiences were that the staff had great skill in ultrasound techniques and managed the examination in a confident way. *They were terrific [in managing the examination] but afterwards, when we came out, we were totally confused (mother and father 9)*. The parents' frustration was caused by the staff's lack of competence concerning the handling of the parents' situation. The staff was expected to be sympathetic, to feel empathy and to consider the parents' perspective in the situation. *Well I don't know how many examinations they are doing every day but (mother 5) yes, it felt more like a production line (father 5)*. Some of the parents found consolation in the statistics concerning the frequency of the diagnosed abnormality presented by the sonographer, others in the way the finding was pointed out and explained. Parents appreciated when the sonographer did not reveal if they were stressed for time, even if the parents understood that there was a time limit for each examination. This aspect created more calm and made the situation manageable for the parents. *All the midwives we have met at the ultrasound department have been very calm and never shown that they were stressed for time; they have been nice (mother 8).*

### Wanting to know

The ultrasound examination was appreciated, mainly because they could visualize their unborn child, but also as a tool for making it possible to discover abnormalities. Parents wanted to know about the ultrasound findings. *If you are in such situation as this you want to know (mother 2)*. One father speculated on the point of knowing even if it was not possible to do anything about the abnormality, and thought the advantages of knowing outweighed the disadvantages. *I think if you can't do anything about the finding, well then it is as it is anyway but if you can do something perhaps you need to discuss different possibilities and views (father 3)*. Some talked about the false sense of security ultrasound examination can give if the result is false negative and how important it is that parents realize this. *However, much you check, you can't decide how things are going to be, because you will never know (mother 4)*. None of the participants in the study said that because of the incident they would not have more children, but they did express that they probably would feel very anxious about a future ultrasound examination, although that would not deter them because they felt it was important. *I can say that I will not look forward to the first ultrasound if we get another baby (mother 5).*

### Needing support

All parents agreed on that support was very important in their situation. The kind of support they preferred or needed differed. Support such as counselling from a specialist physician, consultation with a psychologist or support from a midwife or relatives and friends was emphasised. Many talked about how the specialists had supported them in their trying situation and had experienced this as one of the decisive parts in the process of making their situation manageable. Very few of the parents were offered psychological counselling. *Our message from this event is that we needed someone to talk to, we needed more information both facts but also what will happen after the delivery, what to expect (mother and father 9)*. Sometimes the parents excused this behaviour or routine by saying that the staff probably thought they already had an established contact. One couple described how they thought the situation should be dealt with. *It should be introduced to the parents as "now we will make an appointment with our psychologist because this is our normal routine in a case like this"; it should be more of a statement than a question. It is easy to look upon oneself as weak but this is a normal reaction to an abnormal situation (mother and father 1).*

### Knowledge as a strength

The parents did not only want to know about the ultrasound findings, they thought it was also beneficial for them. It made it possible for them to prepare and to understand the diagnosis and how it would affect their child. The advantages of knowing could be what to expect regarding how the child will look at birth. *I think it is quite good to know because otherwise I think I had lost the joy over the delivery if I had seen our son with these feet which were rotated inwards, not knowing what it was, I think I would have been very shocked, actually (mother 5)*. Making arrangements regarding the birth, possibilities for specialists such as pediatricians to attend during or after the delivery were things the parents embraced with the word "preparation". *If they had not found the heart anomaly during the ultrasound, he had only been able to be outside the uterus for four hours and also not feeling so well (mother 6)*. The strength from knowing was to not having to continue a whole pregnancy, if the child had no chance of surviving outside the uterus. *There wasn't even a drop of amniotic fluid and the lungs ..., so he would never have survived. We received the medical record from the autopsy and then we realized that he had so many abnormalities that it was not possible for him to survive, whatever was done (mother 1)*. Among the parents with an extra, additional diagnosed abnormality after birth, there was an understanding of the fact that it is not possible to detect all abnormalities by an ultrasound examination and therefore they did not feel any disappointment.

### Taking a new direction

Parents who received bad news from their ultrasound examination entered a new phase in their life. Their joyful plans of being a family were now exchanged for anxiety and an uncertainty about how the future for them, as parents, and for their child would turn out. Many parents remarked on the initial shock but how they, after a while and with good support, saw a new day on the horizon. *I don't know when this hero [the physiotherapist] came along and actually solved everything for us, well it only took ten minutes and then we were very calm about the whole situation (father 5)*. With new facts regarding the situation, time was an important ingredient in order to realise the new direction. After this period and with good support from specialist physicians, the parents began to see the situation from a new perspective and adapted to what these new conditions offered. *It felt so well prepared for; they even had a pediatrician present during the delivery who would look at the cleft lip (mother 8)*. It is obvious that how the situation turned out for the parents was dependent on how the specialists who would treat the abnormality and other professionals looked upon the finding, the management of additional tests, planning for the birth, information etc. Some couples had not visited a neonatal care unit or talked to any specialist physician and were, after the delivery, unaware of the routines that awaited them.

## Discussion

Parents have the knowledge that ultrasound examination is a tool for identifying fetal abnormalities [12], even though they might only think about the examination as a confirmation of their pregnancy [17]. The information about abnormal ultrasound findings comes unexpectedly to most parents and creates a feeling of vacillating between emotional confusion and sense of reality. The assessment of the impact of the diagnosis on the wellbeing of the fetus is constantly on their minds. When the parents reach a state of uncertainty and comprehend their expectations are broken, they realize the need to feel cared for and cared about. Of these two, being cared about is the most important. This statement embraces both the fetus and both parents. Whatever is done their anxiety can never be totally prevented, but unnecessary anxiety can be reduced.

The parents experienced the staff as having extensive knowledge and great skill concerning ultrasound techniques and trusted their way of performing the examination. However, a lack in the psycho-social approach leads to the parents feeling they are not being as equally prioritized as the technical management, which is also reported in other studies [10,24]. Even if the parents value and desire a more empathetic approach, they are still aware of the importance of diagnosing the ultrasound findings. This being so, they tend to justify the staff's behaviour and accept their management of the situation.

Other studies [7,18,24,25] have reported that some parents had the feeling that something was wrong even before the midwife informed them about the ultrasound findings. This was due to the body language of the midwife and that she became quiet during the examination. The parents also had a feeling of that the midwife was not permitted to discuss the findings, nor the management of the situation or possible treatment. Similar findings are reported by Lalor et al [24].This handling of the situation is accepted by the parents but on one condition, i.e. they want to talk to a responsible person who is permitted, by their position, to inform them about the findings and furthermore they want this meeting to take place on the same day as the ultrasound examination. This study and previous studies show that prolonged delay can give free scope to the parents' imagination and in turn lead to an unnecessary degree of anxiety [2,7,18].

Even if an abnormality is potentially treatable from a health professional's perspective, parents may not view it in such simplistic terms [24]. This could be the reason why some parents experience that the staff give the impression that they consider that the parents overreact concerning the abnormality. This is a part in the parents feeling of vacillating. The child being in one way unhealthy but on the other hand not so unhealthy, makes the parents consider that perhaps the child is healthy after all. Some parents had the feeling that they were marginalised during the ultrasound examination; it was all about the unborn child. A study from Sweden has also shown the difficulties felt by the sonographer when identifying an abnormality in a routine screening situation, such as feelings of grief and powerlessness, which can, to some extent, explain why the parents experience this feeling [31]. The unborn child is of course important and should be in focus, but for the parents this is a unique situation in which they need to be reassured. If the parents feel well emotionally, this could be beneficial for the child. Teixeira [32] demonstrated that anxiety in pregnancy can lead to higher blood vessel resistance and affect the circulation in the placenta which in turn can lead to fetal growth retardation. All parents feel uncertainty and anxiety, after receiving negative information about the ultrasound findings, irrespective of how severe the abnormality is [26], which even is found by others [24,10]. Initial anxiety becomes intensified due to the environment, the darkness of the examination room and the droning sound from the ultrasound machine. These are circumstances that cannot be avoided, but it is important that the sonographer reflects on how such things influence the parents' situation. Lalor et al [24] found in their interviews that parents were very sensitive towards how bad news is imparted. Narratives in our study describe how women were lying down, half-naked in the dark room and how this made the parents feel vulnerable and at a disadvantage. It is possible to change this aspect by proper management of the situation, based on respect for the parent's vulnerable status.

Similar to a previous study regarding detection of choroid plexus cysts [7], the parents in this study did not think it was possible to have this experience without feeling concerned, but the treatment offered by the caregivers had an impact on how they can manage the situation. They felt secure when encountering other professionals such as obstetricians and other specialist physicians in the field of the given abnormality; this has also been reported in other studies [24,7,10]. None of the interviewed parents were offered psychological counseling. Parents in a situation like this have a need to talk to someone. Not all of the parents need professional counseling, but all have a need to talk about their situation. Perhaps this can send a signal to the parents about how the health care professionals look upon the abnormality and how acceptable it is to be anxious. On the other hand, the Irish study [24] showed that the women who took part preferred to talk to a midwife, since they associated departments of social work or psychiatry with a social stigma.

A theoretical sampling was made and the participants were selected with consideration for the purpose of the study. The procedure does not stand in opposition to grounded theory [30]; as long as it is not a selection made by a predicted idea of what could be important. The couples were interviewed together, due to ethical considerations, and therefore no specific gender perspective has been investigated that probably could have been seen if the parents had been interviewed separately. However, the benefit when interviewing them as a couple was that during many interviews the parents discussed the issue themselves with the interviewer just following their interaction. Furthermore, only Swedish speaking parents were interviewed. The time interval between the ultrasound examination and the interview was long, but there is research that suggests that an experience such as giving birth is something that is well remembered, no matter how many years that have elapsed since the event [33]. It would be reasonable to assume that an abnormal ultrasound also is such a specific occasion that will be well remembered.

In grounded theory the concept "saturation" is central which means that no new information is forthcoming about the study area which will surprise the researcher. All information at this point can be explained by the theory/model. According to Glaser [34] this experience is trustworthy since a theoretical sensitivity for the material is developed during the analysis. Saturation in this study was reached before the last interview. The first author coded all interviews, discussing this with the others during the process. All the authors were involved in the analysis and identification of categories. The third and fourth authors also scrutinised the material, and have extensive experience of performing ultrasound examinations and therefore also have experience of parents' reactions in an ultrasound situation.

In grounded theory the concept "validity" is not a discussed issue. Instead four other concepts are in focus for judging the validity of the material; fit, relevance, workability, and modifiability [29]. The authors agreed upon the *fit *of the categories but also how this had a clinical *relevance*. The implications show the *workability *with the problem and finally the *modifiability *i.e. the possibility to alter the theory when new relevant data is included, which is part of an ongoing process in our research. A grounded theory study can never be right or wrong, only have more or less fit, relevance, workability and modifiability [29].

## Conclusions

Information about the abnormalities found with ultrasound was distressing for the parents. Irrespective of being informed about the abnormality at once or simply informed that something was wrong, made the parents hover between the thought that the fetus had some abnormalities and that the fetus was normal i.e. healthy. The ultrasound examination was described by the parents as a positive and important event, a possibility to connect with their unborn child but also to identify abnormalities. During the scan, environmental factors strengthened the feeling of anxiety when the parents felt that something was different. Though the parents were aware of that the examination could show an abnormality, they anticipated that would be normal. When a deviation was found it was difficult for the parents to understand the information given due to their anxiety. Feelings like being left out, ignored, feeling vulnerable, made it difficult to manage the situation. The sonographers were skilled in ultrasound techniques and in managing the examination but had a lack of competence concerning the handling of the parents' anxiety. Parents wanted to know about the ultrasound findings and the support was very important in their situation but differed. Knowing about the ultrasound findings was also beneficial in preparing and understanding the diagnosis concerning how it affected the child. Joyful plans of being a family were exchanged for anxiety and uncertainty about the future.

Implications based on important factors for supporting the parents' in this context are; their need to receive honest information about the findings and strategy for the management of the abnormality without prolonged delay and while they are sitting in an up-right position; the examiner must be aware of how anxiety can be intensified by environmental factors; all parents ought to be offered someone to talk to - a midwife, a specialist physician or, if necessary, given psychological counseling and this should be a part of the routine.

## Competing interests

The authors declare that they have no competing interests.

## Contribution to authorship

AKL, ECS and AKD designed the study. AKL interviewed most of the parents and AL interviewed two parents. AKL, AL, AKD conducted the data analysis. AKL prepared the first draughts of the paper. All authors read and made substantial comments and contributions to subsequent draughts and approved the final submitted version.
